# Visualization of Intrapulmonary Lymphadenopathy on Vessel-Suppressed Computed Tomography in a Patient With Sarcoidosis

**DOI:** 10.1155/crra/3546986

**Published:** 2025-09-16

**Authors:** Seitaro Ishikawa, Atsushi Takamatsu, Kotaro Yoshida, Miho Okuda, Satoshi Kobayashi

**Affiliations:** Department of Radiology, Kanazawa University Graduate School of Medical Sciences, Kanazawa, Ishikawa Prefecture, Japan

**Keywords:** intrapulmonary lymph node, pulmonary nodule, sarcoidosis, vessel-suppressed CT

## Abstract

We report a case of a 55-year-old woman diagnosed with sarcoidosis involving multiple intrapulmonary lymph nodes that were clearly visualized on vessel-suppressed CT imaging. Intrapulmonary lymph node involvement in patients with sarcoidosis has been reported less frequently and has not been well discussed. This may be attributed to the fact that intrapulmonary lymph nodes present as small nodular lesions located along pulmonary vessels, making them difficult to detect on conventional imaging. Vessel-suppressed CT enhances visualization of pulmonary lesions by selectively suppressing vascular structures. This case highlights the clinical utility of vessel-suppressed CT for improved detection of intrapulmonary lymph node lesions in the diagnosis of sarcoidosis.

## 1. Introduction

Sarcoidosis is a multisystem disease of unknown etiology that causes granuloma formation in various organs [1]. Chest abnormalities in patients with sarcoidosis include hilar and mediastinal lymphadenopathy, micronodules along the lung parenchyma following lymphatic pathways, and fibrosis [2]. Intrapulmonary lymph node involvement in patients with sarcoidosis is less frequently reported and remains poorly characterized [3–6]. This underrepresentation may be attributed to several factors: these lesions may not be recognized as sarcoidosis-related findings, and intrapulmonary lymph nodes typically present as small nodules with a diameter of less than 10 mm located along vascular structures (intrapulmonary lymphatic pathways), making their identification challenging in routine clinical practice [7]. Recently, advanced imaging software utilizing deep learning technology for pulmonary vessel suppression on chest CT has become commercially available. Herein, we report a case of sarcoidosis with multiple intrapulmonary lymph nodes that were clearly visualized on vessel-suppressed CT imaging, demonstrating the clinical utility of this technique for detecting perivascular nodular lesions.

## 2. Case Presentation

**Figure 1:**
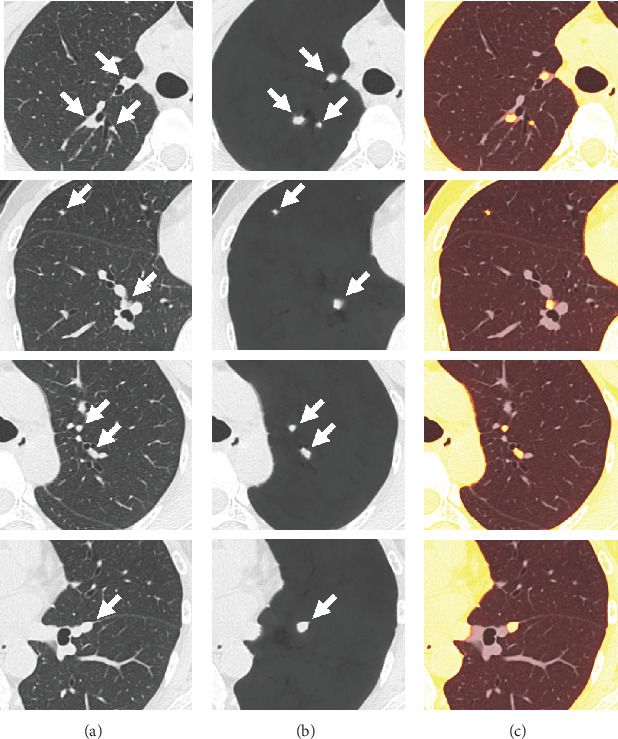
(a) The zoomed-in axial noncontrast CT image (0.6-mm slice thickness) demonstrates multiple nodules along pulmonary vessels in both lungs (arrows). The nodules are in close proximity to existing pulmonary structures, limiting assessment of their morphology and dimensions. (b) On the vessel-suppressed CT (ClearRead CT, Riverain Technologies, Miamisburg, Ohio), multiple nodules along the pulmonary vessels are identified more clearly than in the original images (a) through suppression of existing pulmonary structures. Their morphology and size are easily recognized (arrows). (c) In the fusion image overlaying the original CT (a) and the vessel-suppressed CT (b), the nodules are shown in yellow against the existing lung structures. Multiple nodules are highlighted along the blood vessels and interlobar pleura, indicating intrapulmonary lymph nodes.

**Figure 2:**
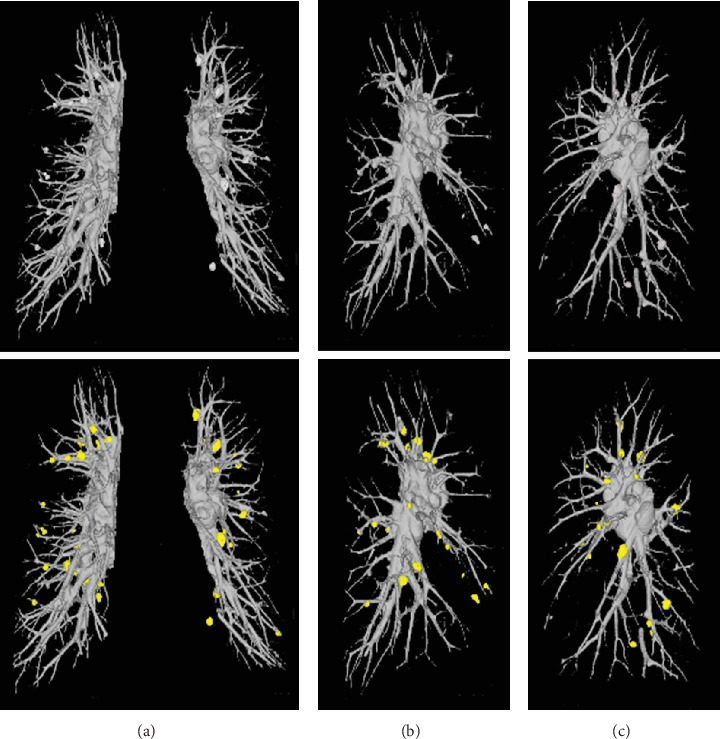
On 3D images created from the CT, pulmonary nodules and pulmonary vessels extracted from the original CT are shown in light gray (upper panels of (a), (b), and (c)). In the fusion images (lower panels), where the original CT and the vessel-suppressed CT are superimposed, the nodules extracted in the vessel-suppression image are shown in yellow. These reconstructions demonstrate the spatial relationship between pulmonary vessels and multiple nodules, providing three-dimensional visualization of nodule distribution. (a) Frontal view of both lungs showing original and fusion images. (b) Lateral view (RAO at 90°) of the right lung displaying original and fusion images. (c) Lateral view (LAO at 90°) of the left lung showing original and fusion images.

A 55-year-old woman who had no specific medical history presented with pain in her right eye and was diagnosed with uveitis. Laboratory tests revealed elevated soluble interleukin-2 receptor levels to 939 U/mL. She had normal angiotensin-converting enzyme levels and normal blood and urinary calcium levels. No elevated antibody titers suggestive of infectious diseases—such as cytomegalovirus, Epstein–Barr virus, tuberculosis, syphilis, or fungal infections—were detected. Chest radiography showed bilateral hilar lymphadenopathy. Unenhanced CT confirmed enlargement of the hilar and mediastinal lymph nodes. Multiple small nodules (3–10 mm in diameter) were suspected in both lungs along lymphatic channels, including the pulmonary vessels and interlobar pleura; however, many nodules were difficult to identify due to their proximity to the pulmonary vessels, appearing as a web-like shape on CT. On vessel-suppressed CT imaging generated by ClearRead CT (Riverain Technologies, Miamisburg, Ohio), in which normal pulmonary vessels and lung parenchyma are suppressed, multiple nodules adjacent to pulmonary vessels were clearly identified (Figures 1 and 2). As a result, a total of 54 pulmonary nodules were clearly visualized (eight in the right upper lobe, 12 in the right middle lobe, nine in the right lower lobe, 17 in the left upper lobe, and eight in the left lower lobe). Gallium-67 scintigraphy revealed uptake in the enlarged bilateral hilar, mediastinal, and right inguinal lymph nodes. No other diseases were detected on systemic evaluation. Based on these findings, the patient was diagnosed with sarcoidosis [8]. The uveitis improved with topical ophthalmic treatment. Electrocardiographic and echocardiographic findings were normal. Because no other organ involvement was observed and the patient remained asymptomatic, no systemic treatment was administered. During follow-up, no new organ lesions or worsening of chest lesions were noted.

## 3. Discussion

Sarcoidosis is a systemic granulomatous disease of unknown etiology. The prevalence of sarcoidosis is elevated in Northern Europeans and African Americans, with a slight female predominance [1]. More than 90% of patients with sarcoidosis have thoracic involvement, and the most common feature in the chest is bilateral hilar and mediastinal lymphadenopathy [1]. Pulmonary lesions in sarcoidosis are characterized by microscopic nodules along lymphatic channels, such as the bronchial perivascular bundles, interlobar septa, interlobar pleura, and subpleural areas. A cluster of these nodules is known as the galaxy sign [2].

Intrapulmonary lymph nodes are anatomical structures located between the hilar lymph nodes and microscopic intrapulmonary lymphatic structures. These macroscopic nodules are less than 10 mm in diameter and are pathologically identified in patients with a smoking history or silicosis exposure. Differentiation of intrapulmonary lymph nodes from primary lung cancer or pulmonary metastases is based on their characteristic distribution along lymphatic channels and polygonal shape [7].

Although the multiple pulmonary nodules in this case were not pathologically confirmed, the patient exhibited both bilateral hilar lymphadenopathy and uveitis, which are characteristic of sarcoidosis. According to the American Thoracic Society clinical practice guidelines, the diagnostic criteria for sarcoidosis were fulfilled in this case [8]. Therefore, the multiple pulmonary nodules were considered to represent intrapulmonary lymph node lesions associated with sarcoidosis. This assessment was based on the nodules' consistency in appearance and distribution with known intrapulmonary lymph nodes, combined with the absence of other causative factors for intrapulmonary lymph node formation, such as smoking or silicosis exposure. Furthermore, the nodules resembled those described in previous reports of multiple intrapulmonary lymph node lesions due to sarcoidosis confirmed by surgical resection [4]. Therefore, our case is considered to represent intrapulmonary lymph node involvement in sarcoidosis. Intrapulmonary lymph node lesions in sarcoidosis are very rare, with only four cases reported [3–6]. The low frequency of these reports may be attributed to the fact that these nodules are frequently overlooked, as they are challenging to distinguish from adjacent pulmonary structures on conventional CT imaging. However, a case of sarcoidosis with only intrapulmonary lymph node lesions, without other organ involvement, has been reported, and awareness of this finding is clinically important [5]. In addition, because the frequency of hilar and mediastinal lymph node enlargement decreases with age [9], it is important to detect intrapulmonary lymph nodes and other lesions in elderly patients.

Cases with multiple pulmonary nodules due to sarcoidosis often pose a diagnostic challenge in differentiating from lung cancer. Pulmonary lesions in sarcoidosis tend to be multiple compared to lung cancer and are typically characterized by smaller size, lower density, and less frequent spiculated morphology [10]. These characteristics are consistent with the present case. However, radiological diagnosis remains challenging in a certain number of patients, and cases of coexisting sarcoidosis and lung cancer have been reported [11, 12]. Vessel-suppressed CT can more clearly delineate the imaging characteristics of nodules compared to conventional CT and may contribute to the differential diagnosis between sarcoidosis and lung cancer. This approach may reduce unnecessary invasive procedures.

In our case, except for some solitary nodules, it was difficult to detect intrapulmonary lymph nodes by separating them from the pulmonary vessels. However, vessel-suppressed CT facilitated clear delineation. The ClearRead CT software was developed to assist radiologists in the interpretation and automatic detection of lung nodules and was approved for use in the United States and Europe in 2016. This imaging process suppresses existing lung structures and improves the visibility of pulmonary nodules by emphasizing them. Although it should be noted that image processing may result in false-positive or false-negative findings at a certain frequency during interpretation, several studies have reported successful nodule detection using vessel-suppressed CT imaging [11–13].

Most nodules exhibited the typical imaging characteristics of intrapulmonary lymph nodes: less than 10 mm in diameter, polygonal or round in shape, and located along lymphatic channels. In particular, the nodules had smooth margins [14]; in contrast, common pulmonary lesions in sarcoidosis display irregular margins due to clustering of microscopic granulomas. Normal intrapulmonary lymph nodes are predominantly located in the lower lobes [7]; however, in this case, the nodules were distributed chiefly in the upper and middle lobes, possibly because pulmonary lesions in sarcoidosis occur more frequently in these regions [15]. Vessel-suppressed CT imaging successfully distinguished nodules abutting intrapulmonary vessels, thereby avoiding misidentification of the vessels themselves. This underscores its value as a diagnostic aid for assessing visually challenging lesions.

## 4. Conclusion

Intrapulmonary lymph node lesions in sarcoidosis are rarely reported, which may be attributed to the difficulty in recognizing these small nodules located along pulmonary vessels on conventional imaging. Vessel-suppressed CT is an imaging technique that selectively suppresses existing pulmonary structures, including vascular elements, and significantly enhances the visualization of multiple intrapulmonary lymph node lesions in this case. This technique enables detection of lesions that are inherently challenging to identify as manifestations of sarcoidosis on standard CT imaging. Furthermore, vessel-suppressed CT may facilitate investigation of the true prevalence and characteristics of the relationship between sarcoidosis and intrapulmonary lymph node involvement.

## Data Availability

The data that support the findings of this study are available from the corresponding author upon reasonable request.

## References

[1] Statement on Sarcoidosis (1999). Joint Statement of the American Thoracic Society (ATS), the European Respiratory Society (ERS) and the World Association of Sarcoidosis and Other Granulomatous Disorders (WASOG) Adopted by the ATS Board of Directors and by the ERS Executive Committee, February 1999. *American Journal of Respiratory and Critical Care Medicine*.

[2] Sève P., Pacheco Y., Durupt F. (2021). Sarcoidosis: A Clinical Overview From Symptoms to Diagnosis. *Cells*.

[3] Ishiyama M., Kawai K., Tawara M. (2017). A case of sarcoidosis discovered following intrapulmonary lymphadenopathy. *Clinical Radiology*.

[4] Nagashima T., Morohoshi T., Yamamoto T., Gorai A., Tsuura Y. (2009). A Case of Sarcoidosis Differentiated From Metastatic Lung Tumor. *Journal of the Japanese Association for Thoracic Surgery*.

[5] Tamagaki G., Matsushita H. (2017). A Case of Sarcoidosis Without Bilateral Hilar and Mediastinal Lymphadenopathy That Was Diagnosed From Multiple Intrapulmonary Lymph Nodes. *Journal of the Japan Society for Respiratory Endoscopy*.

[6] Yasu R., Awai K., Azuma K. (1999). A Case of Intrapulmonary Lymph Node Associated With Sarcoidosis. *Japanese Journal of Clinical Radiology*.

[7] Hyodo T., Kanazawa S., Dendo S. (2004). Intrapulmonary Lymph Nodes: Thin-Section CT Findings, Pathological Findings, and CT Differential Diagnosis From Pulmonary Metastatic Nodules. *Acta Medica Okayama*.

[8] Crouser E. D., Maier L. A., Wilson K. C. (2020). Diagnosis and Detection of Sarcoidosis. An Official American Thoracic Society Clinical Practice Guideline. *American Journal of Respiratory and Critical Care Medicine*.

[9] Sawahata M., Sugiyama Y., Nakamura Y. (2014). Age-Related Differences in Chest Radiographic Staging of Sarcoidosis in Japan. *European Respiratory Journal*.

[10] Catelli C., Guerrini S., D'Alessandro M. (2024). Sarcoid Nodule or Lung Cancer? A High-Resolution Computed Tomography-Based Retrospective Study of Pulmonary Nodules in Patients With Sarcoidosis. *Diagnostics*.

[11] Shin H. J., Kim M. S., Kho B. G. (2020). Delayed Diagnosis of Lung Cancer Due to Misdiagnosis as Worsening of Sarcoidosis: A Case Report. *BMC Pulmonary Medicine*.

[12] Martini K., Blüthgen C., Eberhard M. (2021). Impact of Vessel Suppressed-CT on Diagnostic Accuracy in Detection of Pulmonary Metastasis and Reading Time. *Academic Radiology*.

[13] Yoshida K., Takamatsu A., Toshima F. (2023). Computer-Aided Detection of Subsolid Nodules on Chest Computed Tomography: Assessment of Visualization on Vessel-Suppressed Images. *Journal of Computer Assisted Tomography*.

[14] Nishimura K., Itoh H., Kitaichi M., Nagai S., Izumi T. (1995). CT and Pathological Correlation of Pulmonary Sarcoidosis. *Seminars in Ultrasound, CT, and MR*.

[15] Silva M., Nunes H., Valeyre D., Sverzellati N. (2015). Imaging of Sarcoidosis. *Clinical Reviews in Allergy and Immunology*.

